# American Society of Anesthesiologists’ (ASA) Physical Status System and Risk of Major Clavien-Dindo Complications After Robot-Assisted Radical Prostatectomy at Hospital Discharge: Analysis of 1143 Consecutive Prostate Cancer Patients

**DOI:** 10.1007/s13193-022-01577-9

**Published:** 2022-07-13

**Authors:** Antonio Benito Porcaro, Riccardo Rizzetto, Nelia Amigoni, Alessandro Tafuri, Alberto Bianchi, Sebastian Gallina, Rossella Orlando, Emanuele Serafin, Alessandra Gozzo, Clara Cerrato, Giacomo Di Filippo, Filippo Migliorini, Stefano Zecchini Antoniolli, Giovanni Novella, Vincenzo De Marco, Matteo Brunelli, Maria Angela Cerruto, Enrico Polati, Alessandro Antonelli

**Affiliations:** 1 Present Address: Department of Urology, University of Verona, Azienda Ospedaliera Universitaria Integrata, Verona, Italy; 2 Department of General and Hepatobiliary Surgery, University of Verona, Azienda Ospedaliera Universitaria Integrata, Verona, Italy; 3 Department of Pathology, University of Verona, Azienda Ospedaliera Universitaria Integrata, Verona, Italy; 4 Department of Anesthesiology, University of Verona, Azienda Ospedaliera Universitaria Integrata, Verona, Italy

**Keywords:** Prostate cancer, Radical prostatectomy, American Society of Anesthesiologists’ (ASA), Physical status system classification, Robot-assisted radical prostatectomy, Postoperative complications, Clavien-Dindo grading complications system

## Abstract

**Objective:**

To test the hypothesis of associations of preoperative physical status system with major postoperative complications at hospital discharge in prostate cancer (PCa) patients treated with robot-assisted radical prostatectomy (RARP).

**Materials and Methods:**

In a period ranging from January 2013 to October 2020, 1143 patients were evaluated. The physical status was assessed by the American Society of Anesthesiologists’ (ASA) system, which was computed trained anesthesiologists. The Clavien-Dindo system was used to classify postoperative complications, which were coded as major if greater than 1.

**Results:**

ASA physical status system included class I in 102 patients (8.9%), class II in 934 subjects (81.7%), and class III in 107 cases (9.4%). Clavien-Dindo complications were distributed as follows: grade 1: 141 cases (12.3%), grade 2: 108 patients (9.4%), grade 3a: 5 subjects (0.4%), grade 3b: 9 patients (0.8%), and grade 4a: 3 cases (0.3%). Overall, major complications were detected in 125 cases (10.9%). On multivariate analysis, major Clavien-Dindo complications were predicted by ASA score grade II (adjusted odds ratio, OR = 2.538; 95%CI 1.007–6.397; *p* = 0.048) and grade III (adjusted OR 3.468; 95%CI 1.215–9.896; *p* = 0.020) independently by pelvic lymph node dissection (PLND) and/or blood lost.

**Conclusion:**

In RARP surgery, the risk of major postoperative Clavien-Dindo complications increased as the physical status system deteriorated independently by performing or not a PLND and/or large intraoperative blood lost. The ASA score system was an effective predictor of major Clavien-Dindo complications, which delayed LOHS in RARP surgery. Confirmatory studies are required.

**Supplementary Information:**

The online version contains supplementary material available at 10.1007/s13193-022-01577-9.

## Introduction

Actually, clinically localized prostate cancer (PCa) is a priority health problem for its prevalence all over the world [1, 2]. Stratification of clinical PCa into risk groups has prognostic relevance with impact on treatment options that include active surveillance, watchful waiting, radical prostatectomy (RP), and radiotherapy (RT), as well [1, 2]. Surgery contemplates removal of the prostate gland and associates with removal of loco-regional pelvic lymph nodes (PLND), when recommended [1, 2]. Surgery may be delivered three main approaches, which include video laparoscopy (VLRP), robot-assisted (RARP), and open (ORP) RP according to the surgeon’s expertise [1, 2]. Patients should also be informed that no surgical approach has clearly shown superiority for functional or oncological results, but the strength of the recommendation is weak [1]. In high-risk disease, combinations of surgery, radiotherapy, and androgen blockade are planned by multidisciplinary teams [1, 2].

Guidelines recommend close attention of overdiagnosis and overtreatments on life expectancy and health status for disease specific mortality does not improve when life expectancy is less than 10 years and baseline physical status is impaired by comorbidities [1, 2]. Although surgery and RT are both equivalent for oncological results, a recent Italian multicenter observational study on treatment options for localized PCa, has shown that younger and healthier patients are more likely to undergo RP with RARP being the preferred approach by patients who report better results when compared with both the open and laparoscopic approach [3, 4]. Although RARP with or without PLND is the most frequently performed procedure in high volume centers, the risk of complications is an issue to be discussed when counselling patients [1, 2].

So far, life expectancy, comorbidities and complications are important features that might impact negatively on quality of life, as suggested by guidelines [1, 2]. In patients undergoing RARP with or without PLND, the ability of the patient to withstand stresses related to anesthesia and surgery dependents on actual physical status. The way a healthy patient withstand such stresses is completely different when compared with a subject who is severely ill. As such, we can perform the same anesthesia and operation but the perioperative risks are completely different for we are operating in two completely different physical status systems [5, 6]. The American Association of Anesthesiologists’ (ASA) physical classification system still represents an important tool for evaluating preoperatively the physical status of the patients [1, 2, 5, 6]. Patients elected to RARP are prepared and evaluated preoperatively by trained anesthesiologists in order classify the actual physical status for assessing the operative risk. We wanted to test the hypothesis of associations of the ASA grading system with postoperative complications in patients undergoing RARP surgery.

## Materials and Methods

### Patients and Methods

The study was retrospective and approved by internal Institutional Review Board. Informed signed consent was obtained by all patients. Data were collected prospectively, but evaluated retrospectively. In a period ranging from January 2013 to October 2020, 1143 consecutive patients, who underwent RARP, were included after excluding cases who were under androgen blockade and/or had prior treatments for PCa. Clinical features including age (years), body mass index (BMI; kg/m^2^), PSA (ng/mL), prostate volume (PV, mL), and biopsy positive cores (BPC; percentage) were evaluated. Tumors were staged according to the clinical and pathological TNM system [1]. RARP was eventually associated with PLND according to guideline recommendations or tumor upgrading probability for the low risk category [1, 2, 7]. Lymph node dissection was developed according to a standard anatomical template including external iliac, obturator, Cloquet’s and Marcille’s regions [8, 9]. Since January 2017, our policy is not to place a drain in the pelvic cavity independently by performing or not an extended PLND [10]. Specimens were evaluated for tumor grade and stage, surgical margins, number of removed and metastatic lymph nodes. Tumors were graded according to the International Society of Urological Pathology (ISUP) system [1, 2, 11]. Surgical procedures were performed by 5 skilled and dedicated surgeons of whom two were classified as high volume.

Preoperative physical status system was assessed according to the ASA system that was computed by a dedicated team of anesthesiologists who were referent for the urological department and included consultant and senior trained residents, as well. All patients had been codified a physical status before surgery according to the actual ASA physical status system classification, which is reported in supplementary Table S1. Postoperative surgical complications were classified according to the Clavien-Dindo system [1, 2]. Postoperative complications were graded at hospital discharge and monitored for a period of 90 days. Length of hospital stay (LOHS) was measured in days and started from the day of operation. Hospital readmission events were also evaluated and complications were reclassified according the Clavien-Dindo system [1, 2, 12].

### Study Design and Statistical Methods

The study wanted to test the hypothesis of associations of the ASA physical status system with postoperative complications at hospital discharge. Factors were grouped into physical (ASA, age, BMI), cancer (PSA, BPC, cT, biopsy ISUP, pT, pN, pathological ISUP, and surgical margin status) and perioperative parameters (PLND, operating time, blood lost, and postoperative Clavien-Dindo complications). According to their distributions, continuous variables were represented as medians with relative interquartile ranges (IQR) while categorical factors were assessed as frequencies (percentages). Associations of the ASA system with all parameters, which also included postoperative complications, were assessed by the multinomial logistic regression model (univariate analysis). Clavien-Dindo complications were coded as major (greater than 1) versus minor (up to 1) and associations were investigated by the binomial logistic regression model (univariate and multivariate analysis). The validity of the multivariate model was evaluated by the method of Hosmer–Lemeshow with contingency tables and relative test. The software used to run the analysis was IBM-SPSS version 26. All tests were two-sided with *p* < 0.05 considered to indicate statistical significance.

## Results

**Fig. 1:**
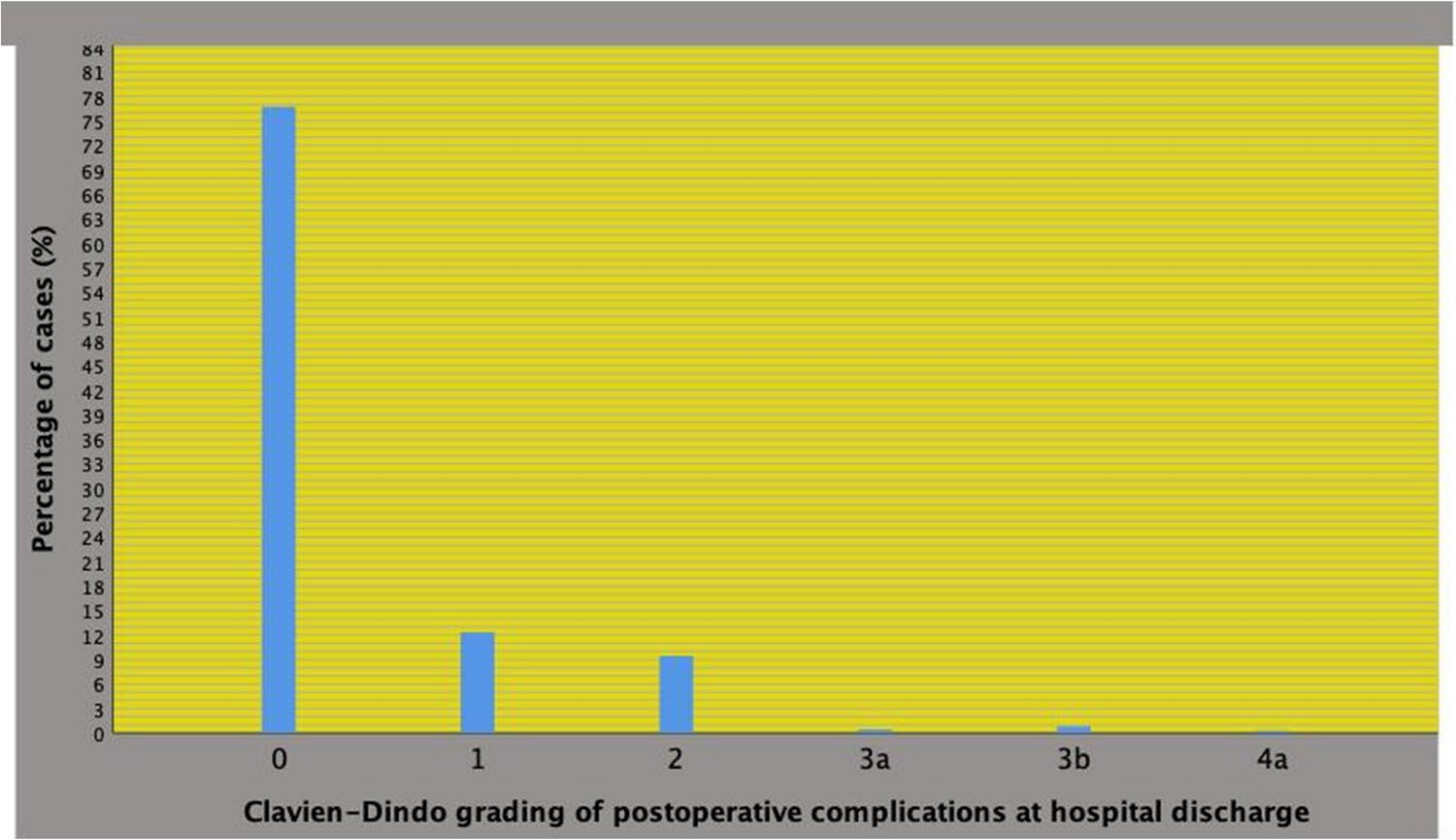
Postoperative Clavien-Dindo complications at hospital discharge in 1143 patients treated with robot-assisted radical prostatectomy (RARP). Complications were distributed as follows: grade 1: 141 cases (12.3%), grade 2: 108 patients (9.4%), grade 3a: 5 subjects (0.4%), grade 3b: 9 patients (0.8%), and grade 4a: 3 cases (0.3%). Overall, major complications (greater than 1) were detected in 125 cases (10.9%)

- *Demographics of the PCa Population*
  The demographics of the patient population is reported in Table 1. All risk groups were represented with the high-risk class including 19.8% of cases and the intermediate risk group 53% of subjects. PLND was performed in 61.2% of cases who had a median number of 25 lymph nodes removed. Postoperative complications at hospital discharge occurred in 266 patients (23.3%) and the distribution, which is illustrated in Fig. 1, according to the Clavien-Dindo system was as follows: grade 1: 141 cases (12.3%), grade 2: 108 patients (9.4%), grade 3a: 5 subjects (0.4%), grade 3b: 9 patients (0.8%) and grade 4a: 3 cases (0.3%). Overall, 125 patients had a Clavien-Dindo complication greater than 1 (10.9%) at hospital discharge.
- *Associations of the ASA Grading System with Physical and Perioperative Factors*

**Table 1:** Demographics of the prostate cancer population (n = 1143) that was treated with robot assisted radical prostatectomy (RARP)

	Median (IQR) or frequency (%)
***Clinical factors***	
Age (years)	65 (60—70)
Body mass index, BMI (kg/m^2)	25,9 (23,9—28)
Prostate specific antigen, PSA (ug/L)	6,5 (4,9—8,9)
Prostate volume, PV (mL)	40 (30—50)
Biopsy positive cores, BPC (%)	29 (17—49)
International Society of Urologic Pathology (ISUP) tumor grade system	
ISUP = 1	444 (38,8)
ISUP = 2	366 (32)
ISUP = 3	197 (17,2)
ISUP = 4	111 (9,7)
ISUP = 5	25 (2,2)
Tumor clinical stage (cT)	
cT1	697 (61)
cT2/3	446 (39)
Clinical nodal stage (cN)	
cN0	1085 (94,9)
cN1	58 (5,1)
D'Amico risk groups	
Low risk class	311 (27,2)
Intermediate risk class	606 (53)
High risk class	226 (19,8)
***Pathological factors***	
Prostate weight; gr (PW)	51 (42—65)
ISUP = 1	144 (12,6)
ISUP = 2	449 (39,3)
ISUP = 3	313 (27,4)
ISUP = 4	161 (14)
ISUP = 5	76 (6,6)
Pathological tumor stage (pT)	
pT2	896 (78,4)
pT3a	112 (9,8)
pT3b	135 (11,8)
Positive surgical margin (PSM)	
no	859 (75,2)
yes	284 (24,8)
Pathological nodal staging (pN)	
pN0	616 (53,9)
pN1	83 (7,3)
pNx	444 (38,8)
Lymph nodes removed (number)	25 (20—32)
***Perioperative factors***	
Surgeon	
high volume surgeon (HVS)	616 (53,9)
low volume surgeon (LVS)	488 (42,7)
unknown	39 (3,4)
Pelvic lymh node dissection (PLND)	699 (61,2)
Operating time; minutes (OT)	233 ((205—259)
Blood lost; mL (BL)	300 (150—400)
Post-operative Clavien-Dindo complication at discharge (CDC)	
grade 1	141 (12,3)
grade > 1	125 (10,9)
Length of hospital stay; days (LOHS)	4 (4—5)
Hospital readmission; n (%)	35 (4,7)

Legend: IQR, interquartile range; %, percentage

**Fig. 2:**
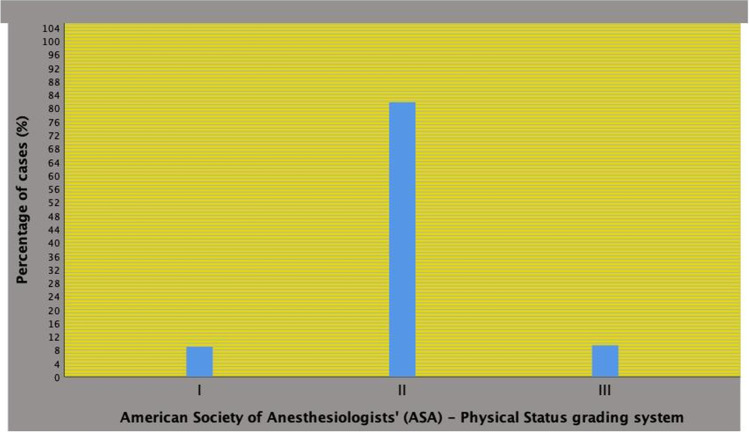
American Society of Anesthesiologists’ (ASA) physical status system in 1143 prostate cancer patients who underwent robot-assisted radical prostatectomy (RARP). ASA system was distributed as follows: class I in 102 patients (8.9%), class II in 934 subjects (81.7%), and class III in 107 cases (9.4%). Overall, major complications (Clavien-Dindo score greater than 1) were detected in 125 cases (10.9%)

The distribution of the ASA physical status system, which is depicted in Fig. 2, was as follows: ASA I in 102 patients (8.9%), ASA II in 934 subjects (81.7%) and ASA III in 107 cases (9.4%). As shown in Table 2, there were significant associations of the ASA grading system with physical and perioperative features. As the physical system deteriorated, age and BMI increased as well the rates of major Clavien-Dindo complications (greater than 1), which delayed LOHS. The distribution of Clavien-Dindo complications greater than 1 were related to the ASA system as follows ASA I: 5 cases (4.9%), ASA II: 104 cases (11.1%), and ASA III: 16 patients (15.0%). Considering other factors, major Clavien-Dindo complications were also increased by PLND as well as blood lost, as shown in Table 3.

**Table 2:** Associations of clinical, pathological and perioperative factors with the American Society of Anesthesiologists' (ASA) Physical Status Classification System in 1143 prostate cancers patients treated with robot assisted radical prostatectomy (univariate analysis)

	ASA I	ASA II	ASA III	ASA II vs ASA I		ASA III vs ASA I		ASA III vs ASA II	
*Statistics*	*Median (IQR) or frequency (%)*	*Median (IQR) or frequency (%)*	*Median (IQR) or frequency (%)*	*OR (95%CI)*	*p-value*	*OR (95%CI)*	*p-value*	*OR (95%CI)*	*p-value*
Age	60 (56—65,1)	65 (61—70)	67 (62—71)	1,103 (1,069—1,137)	< 0,0001	1,131 (1,084—1,181)	< 0,0001	1,026 (0,993—1,055)	0,126
BMI	24,8 (23,6—26,7)	25,9 (23,9—28)	27,7 (24,5- 29,7)	1,126 (1,050—1,207)	0,001	1,304 (1,192—1,426)	< 0,0001	1,158 (1,089—1,232)	< 0,0001
PSA	6,5 (5,1—9,3)	6,5 (4,9—8,9)	6,3 (5—9,1)	1,005 (0,974—1,036)	0,758	1,008 (0,971—1,046)	0,691	1,003 (0,978—1,028)	0,825
PV	38 (30—49,1)	40 (30—50)	40 (32—54)	1,005 (0,992—1,017)	0,462	1,009 (0,994—1,025)	0,231	1,005 (0,994—1,016)	0,376
BPC	29 (17—50)	29 (17—47)	29 (18,7—47)	1,001 (0,992—1,011)	0,805	1,003 (0,991—1,016)	0,587	1,002 (0,993—1,011)	0,625
ISUP < 3	76 (74,5)	664 (71,1)	70 (65,4)	Ref		Ref		Ref	
ISUP > 2	26 (25,5)	270 (28,9)	37 (34,6)	1,189 (0,745—1,896)	0,469	1,545 (0,850—2,808)	0,154	1,300 (0,852—1,984)	0,224
cT < 2	72 (70,6)	568 (60,8)	57 (53,3)	Ref		Ref		Ref	
cT > 1	30 (29,4)	366 (39,2)	50 (39,2)	1,546 (0,990—2,415)	0,055	2,105 (1,190—3,725)	0,011	1,361 (0,911—2,035)	0,132
cN0	97 (95,1)	889 (95,1)	99 (92,5)	Ref		Ref		Ref	
cN1	5 (4,9)	45 (4,8)	8 (7,5)	0,956 (0,696—1,456)	0,835	1,124 (0,641—1,972)	0,684	1,175 (0,774—1,784)	0,448
PW	50 (41—63)	51 (42—65)	55 (45—69,6)	1,004 (0,992—1,015)	0,516	1,012 (0,998—1,026)	0,106	1,008 (0,998—1,018)	0,110
ISUP < 3	55 (53,9)	488 (52,2)	50 (46,7)	Ref		Ref		Ref	
ISUP > 2	47 (46,1)	446 (47,8)	57 (53,3)	1,069 (0,710—1,611)	0,784	1,334 (0,774—2,218)	0,299	1,247 (0,835—1,862)	0,280
pT2	85 (83,3)	737 (78,9)	74 (69,2)	Ref		Ref		Ref	
pT3a	8 (7,8)	88 (9,4)	16 (15)	1,269 (0,595—2,707)	0,538	2,297 (0,930—5,674)	0,071	1,811 (1,010—3,247)	0,046
pT3b	9 (8,8)	109 (11,7)	17 (15,9)	1,397 (0,683—2,858)	0,360	2,170 (0,913—5,158)	0,080	11,553 (0,884—2,731)	0,126
No PSM	69 (67,6)	707 (75,7)	83 (77,6)	Ref		Ref		Ref	
PSM	33 (32,4)	227 (24,3)	24 (22,4)	0,671 (0,432—1,043)	0,077	0,605 (0,327—1,118)	0,109	0,901 (0,558—1,453)	0,668
pN0	616 (53,9)	501 (53,6)	59 (55,1)	Ref		Ref		Ref	
pN1	7 (6,9)	66 (7,1)	10 (9,3)	1,054 (0,461—2,409)	0,901	1,356 (0,483—3,809)	0,563	1,287 (0,628—2,637)	0,491
pNx	39 (38,2)	367 (39,3)	38 (35,5)	1,052 (0,684—1,618)	0,818	0,925 (0,519—1,647)	0,791	0,879 (0,572—1,351)	0,557
LN (n)	27 (20—33)	25 (20—32)	26 (19,5—31,5)	0,982 (0,959—1,005)	0,118	0,978 (0,947—1,010)	0,181	0,996 (0,971—1,022)	0,781
No PLND	39 (38,2)	367 (39,3)	38 (35,5)	Ref		Ref		Ref	
PLND	63 (61,8)	567 (60,7)	69 (64,5)	0,956 (0,628—1,456)	0,835	1,124 (0,641—1,972)	0,684	1,175 (0,774—1,784)	0,448
OT	220 (195—248)	234 (205—260)	238 (206,5—264,5)	1,005 (1,001—1,009)	0,012	1,005 (1,000—1,010)	0,043	1,000 (0,997—1,004)	0,886
BL	255 (150—400)	300 (150—400)	300 (150—425)	1,001 (1,000—1,002)	0,121	1,001 (1,000—1,0029	0,073	1,000 (1,000—1,001)	0,431
LOHS	4 (4—5)	4 (4—5)	4 (4—5)	1,265 (1,050—1,524)	0,013	1,347 (1,103—1,644)	0,004	1,064 (0,980—1,156)	0,140
Clavien-Dindo									
Grade 0	88 (86,3)	705 (75,5)	84 (78,5)	Ref		Ref		Ref	
Grade 1	9 (8,8)	125 (13,4)	7 (6,5)	1,734 (0,851—3,553)	0,130	0,815 (0,290—2,287)	0,697	0,470 (0,212—1,040)	0,062
Grade > 1	5 (4,9)	104 (11,1)	16 (15)	2,596 (1,030—6,543)	0,043	3,352 (1,176—9,558)	0,024	1,291 (0,728—2,290)	0,382

Legend: IQR, interquartile range; OR, odds ratio; CI, confidence interval; see also Table 1

**Table 3:** Associations of clinical, pathological and perioperative factors with Clavien-Dindo Score (CDS) postoperative complications at hospital discharge in 1143 prostate cancer patients treated with robot assisted radical prostatectomy (RARP)

	**CDS < 2**	**CDS > 1**	**CDS > 1 vs CDS < 2**		**CDS > 1 vs CDS < 2**	
	*Statistics*		*Univariate analysis*		Multivariate analysis (*)	
	*Median (IQR) or frequency (%)*	*Median (IQR) or frequency (%)*	*OR (95%CI)*	*p-value*	*OR (95%CI)*	*p-value*
*N (%)*	*1018 (89,1)*	*125 (10,9)*				
Age	65 (61—70)	66 (62—70)	1,021 (0,992—1,051)	0,157		
BMI	25,7 (25,7—28,1)	25,9 (23,9—28,2)	1,016 (0,958—1,077)	0,598		
PSA	6,9 (5,1—9,6)	7 (5,1—11,7)	1,013 (0,994—1,032)	0,189		
PV	40 (30—51)	39,5 (30	1,003 (0,993—1,013)	0,583		
BPC	36 (21,2—53)	33,2 (21—57,3)	1,005 (0,996—1,013)	0,266		
ISUP < 3	725 (71,2)	85 (68)	Ref			
ISUP > 2	293 (28,8)	40 (32)	1,164 (0,781—1,736)	0,455		
cT < 2	627 (61,6)	70 (56)	Ref			
cT > 1	391 (38,4)	55 (44)	1,260 (0,966—1,833)	0,227		
cN0	966 (94,9)	119 (95,2)	Ref			
cN1	52 (5,1)	6 (4,8)	0,937 (0,394—2,227)	0,882		
PW	51 (42—64)	52,2 (43,8—67)	0,998 (0,988—1,008)	0,732		
ISUP < 3	533 (52,4)	60 (48)	Ref			
ISUP > 2	485 (47,6)	65 (52)	1,191 (0,821—1,727)	0,358		
pT2	801 (78,7)	95 (76)	Ref			
pT3a	102 (10)	10 (8)	0,827 (0,417—1,637)	0,585		
pT3b	115 (11,3)	20 (16)	1,466 (0,827—2,467)	0,149		
No PSM	776 (76,2)	83 (66,4)	Ref		Ref	
PSM	242 (23,8)	42 (33,6)	1,623 (1,090—2,416)	0,017	1,437 (0,955—2,164)	0,082
pN0	535 (88,4)	81 (86,2)	Ref			
pN1	70 (11,6)	13 (13,8)	1,227 (0,649—2,318)	0,529		
LN (n)	25 (20—32)	26 (21—32)	1,002 (0,981—1,024)	0,828		
No PLND	413 (40,6)	31 (248)	Ref		Ref	
PLND	605 (59,4)	94 (75,2)	2,070 (1,354—3,165)	0,001	2,048 (1,239—3,383)	0,005
HVS	557 (56,7)	59 (48,4)	Ref			
LVS	426 (43,3)	62 (51,2)	1,374 (0,941—2,005)	0,100		
OT	231 (205—257,5)	245,5 (205—270)	1,006 (1,003—1,010)	< 0,0001	1,001 (0,997—1,006)	0,521
BL	300 (150—400)	300 (200—400)	1,001 (1,000—1,001)	< 0,0001	1,001 (1,000—1,001)	< 0,0001
LOHS	4 (4—5)	6 (4,7—9)	1,654 (1,489—1,839)	< 0,0001		

Legend: IQR, interquartile range; OR, odds ratio; CI, confidence interval; see also Table 1; HVS, high volume surgeon; LVS, low volume surgeon; (*): LOHS not included in the model beacuse it is a consequence of postoperative complications

c) Association of the ASA Grading system with the Risk of Major Postoperative Complications at Hospital Discharge.

As shown in Table 4, the risk of major Clavien-Dindo complications (greater than 1) were increased by ASA score grade II (adjusted odds ratio, OR = 2.538; 95%CI 1.007–6.397; *p* = 0.048) as well as by ASA grade III (adjusted OR 3.468; 95%CI 1.215–9.896; *p* = 0.020) independently by PLND (OR = 2.102; 95%CI 1.372–3.221; *p* = 0.001) and blood lost above the third quartile, which was 400 mL (OR = 1.619; 95%CI 1.025–2.537; *p* = 0.039). The fit of the model was assessed through contingency tables including 5 groups by the test of Hosmer–Lemeshow (chi-squared 0.597; degrees freedom = 3; *p* = 0.897), which indicated that the predictive model did fit quite well; moreover, overall model accuracy resulted 89.1%.

**Table 4:** Multivariate analysis of factors associated with the risk of postoperative Clavien-Dindo Score (CDS) complications greater than one at hospital discharge in 1143 prostate cancer patients treated with robot assisted radical prostatectomy (RARP)

Multivariate model						
	Total	CDS < 2	CDS > 1	CDS > 1 vs CDS < 1		
	*n*	*n (%)*	*n (%)*	OR (95% CI)		*p—value*
ASA I	102	97 (95,2)	5 (4,9)	Ref		
ASA II	934	830 (88,9)	104 (11,1)	2,538 (1,007—6,397)		0,048
ASA III	107	91 (85)	16 (15)	3,468 (1,215—9,896)		0,020
No PLND	444	413 (93)	31 (7%)	Ref		
PLND	699	605 (86,6)	94 (13,4%)	2,102 (1,372—3,221)		0,001
BL up to 400 mL	953	857 (89,9)	96 (10,1)	Ref		
BL > 400 mL	190	161 (84,7)	29 (15,3%)	1,613 (1,025—2,537)		0,039
Assessing the fit of the model (*)						
		CDS < 2		CDS > 1		
Group	Total	Observed	Predicted	Observed	Predicted	
1	98	93	93,7	5	4,3	
2	299	279	279,5	20	19,5	
3	108	99	97,7	9	10,3	
4	471	413	410,9	58	60,1	
5	167	134	136,2	33	30,8	
Total	1143					

Legend: OR, odds ratio; CI, confidence interval; see laso Table 1; (*), test of Hosmer-Lomeshow: chi-squared 0,597; degree freedom = 3; p = 0,897

## Discussion

In 1941, the American Society of Anesthesiologists’ (ASA) introduced the physical classification system, which was independent from variables dependent on anesthesiology and surgery, in order to assess the ability of patients to withstand surgery through anesthesiology [5]. In 1961, Dripps and associates showed that the ASA system associated with the risk of mortality related to anesthesiology [6]. As Dripps explained in his paper, the state of anesthesia and the performance of an operation are stresses, and the patient, healthy or desperately ill, must call on reserves to withstand these states [6]. The authors investigated on perioperative deaths, within 30 days since surgery, in approximately 120,000 patients who were evaluated by the ASA grading system, which ranged from 1 to 5 [6]. In this study, it was found out that the ASA system associate with the number of deaths related to anesthesia, which were 1285, specifically, as the physical condition of the patient worsened, anesthesia-related deaths increased [6]. As reported in supplementary Table S1, the actual ASA physical grading system, since the first formulation, includes a further class coded as grade VI, which indicates a declared brain-dead patient whose organs are being removed for donor purposes. In further studies investigating on this subject, the ASA grading system was also an effective index for assessing the surgical operative risk since it correlated to overall surgical mortality [13, 14]. So far, the ASA system became a predictor of mortality risk of both anesthesiology and surgery [14]. Over time, it has been demonstrated that the ASA system through reference examples was reproducible by anesthesia-trained and no anesthesia-trained clinicians [15]. Actually, the ASA score system is used worldwide not only by anesthesiologists, but also by other clinicians [15, 16].

Classifying patients preoperatively according to their physical status and postoperatively at hospital discharge by the Clavien-Dindo classes is pivotal in actual RARP surgery practice. The European Association of Urology (EAU) recommend to include the ASA system and to grade postoperative complications according to the Clavien-Dindo system in order to reduce misleading reports, which have been detected in 35.3% of papers [17]. However, these features are rarely observed in contemporary academic series reporting on outcomes for RARP surgery. In a large study including 2159 PCa patients who underwent RARP between 2005 and 2015, Pompe et al. reported that overall postoperative complications rates were 19.0% including 11.1% as grade I, 6.8% as grade II, 2.3% as grade IIIa, 1.8 as grade IIIb and 0.8% as grade IV with Clavien-Dindo grades greater than two considered as severe complications; however, preoperative evaluation of the physical status was evaluated by the Charlson comorbidity index and not by the ASA system; moreover, associations of the physical status system with complications at hospital discharge were not investigated in the RARP subgroup [18]. Xia et al. while investigating on pre-discharge predictors of readmissions and post discharge complications in RARP cases, found out that preoperative ASA demographics were grade I–II in 6479 cases (64.9%) and grade III–IV in 3496 patients (35.1%); furthermore, readmission rates occurred more frequently in ASA III–IV cases; on multivariate analysis, ASA score III–IV, operating time and LOHS were independent predictors of hospital readmission; however, they did investigate on factors predicting the risk of postoperative Clavien-Dindo complications at hospital discharge [19]. Aning et al., while evaluating 3196 high-risk patients who underwent RP with RARP being the most prevalent approach (60.7%), found out that RARP postoperative “high-grade” complications (Clavien-Dindo score greater than II) were 2.0%; however, data relative to ASA and associations with the risk of postoperative complications in the robotic subgroup were not reported; moreover, only 33.8% of all patients including the open, laparoscopic, and robot-assisted approach did receive PLND, which was performed according to an extended pattern in only 39.0% of cases [20]. Pereira et al. while studying perioperative morbidity and mortality among 35,968 men of the National Surgical Quality Improvement Program (NSQIP) undergoing RP, reported that ASA distribution was 4% for grade I, 61% for grade II, 34% for grade III, and 0.9% for grade IV; furthermore, ASA distributions positively correlated with age, which associated with postoperative complications, with readmission rates and with perioperative mortality; however, stratification according to the surgical approach was not performed as well as assessment of postoperative complications according to the Clavien-Dindo system [21].

Wallerstedt et al. investigated on readmission rates after surgery (RARP or ORP) in a Sweden multicenter trial; in 2764 RARP cases, distribution of ASA system was 63.0% for grade I, 35.0% for grade II and 2.0% for grade III; furthermore, the distribution of RARP postoperative Clavien-Dindo complications included grade I (1.4%), grade II (6.8%), grade IIIa (1.3%), grade IIIb (1.5%), and grade IV (0.14%); however, PLND was performed in only 9.0% of cases and associations of physical status with postoperative complications were not investigated [22]. Oderda et al. investigated on indications and complications of RP associated with PLND in a large European multicenter trial including 12,009 patients who were classified as ASA grade I (22.7%), grade II (65.4%), grade III (11.8%), and grade IV (0.1%); the authors found out that lymph node metastases were more frequently detected in ASA III cases, but less frequently in ASA I patients; however, stratification according to the surgical approach was not performed and associations between ASA score and complications were not investigated; furthermore, the cohort was dated since starting from 1992 and for including several European centers [23]. In a series of 625 Japanese RARP cases, it was found out that only PLND increased the risk of any perioperative complication classified according to the Clavien-Dindo system; however, preoperative physical status of patients was not evaluated [24]. Knipper et al., while evaluating the National Inpatient Sample (NIS) data base for both open and robot-assisted radical prostatectomies in North America, have shown that obesity predicted unfavorable perioperative complications as well as increased total hospital charges at RARP, which included 53,636 cases; however, the study had several limitations because it was retrospective, complications were not graded according to the Clavien-Dindo system, the performance as well as ASA status were not evaluated; furthermore, the trial could not be adjusted for androgen derivation and prior treatments for PCA, as well [25]. In our study, ASA score distribution rates, which are depicted in Fig. 2, were detected in a contemporary cohort that was operated in a tertiary high-volume center including all clinical risk classes. RARP surgery was performed in ASA groups I through III with grade group II being the most represented category. As the ASA grading system deteriorated, the risk of major Clavien-Dindo complications (greater than 1) increased and delayed LOHS. So far, the ASA system was a predictor of major Clavien-Dindo complications, which delayed LOHS, independently by performing a PLND and/or intraoperative blood lost larger than 400 mL in RARP surgery; as a result, major Clavien-Dindo complications delayed LOHS, which impacted on hospital costs. In our cohort, a not negligible group was represented by the ASA III category. Performing surgery in the ASA III category is a hazard and, as such, it should be performed in tertiary referral centers for including dedicated intense care units. Compared with PLND and intraoperative blood lost, the ASA score was by far the strongest predictor of major postoperative complication at hospital discharge, as shown in Table 4. To the best of our knowledge, this is the first study demonstrating that the ASA system is an effective tool for evaluating preoperatively PCa patients undergoing RARP surgery with or without PLND. Furthermore, these results were evaluated in large patient population including all clinical risk classes.

Our study showed associations between physical status features and ASA system in PCa patients undergoing RARP. As the physical system of patients deteriorated, age, BMI, and risk of major Clavien-Dindo complications at hospital discharge increased. These associations may be explained by considering the pathophysiology of the stress system, which is not the same along the ASA groups, that is called to withstand the impact of both anesthesia and surgery on the physical system. Higher levels of stress, increased BMI, surgical approach are features impacting on major complications, which will delay LOHS with drawbacks on both economical and biological costs [26–29]. Indeed, along the hypothalamic pituitary adrenal axis, pathways of the stress system are triggered to a lesser degree by RARP compared with ORP; as such, postoperative recovery is faster for the former [28].

Our study has implications in clinical practice. In patients elected to RARP surgery, the risk of major postoperative complications, which delay LOHS, increase as the ASA system deteriorates. Furthermore, independently by PLND and/or amounts of intraoperative blood lost, the ASA III subgroup is high risk group for the impaired physical system as well as for reduced tolerance to stress triggered by anesthesia and surgery. This represent an important issue when counselling PCa patients who are also considering other treatment options. RARP surgery in the ASA score III category is a hazard a should be performed in appropriate centers in order to manage major postoperative complications.

Our study has limits. First, it was retrospective with relative biases. Second, operations were not performed by a single surgeon. Third, ASA score were computed by several resident anesthesiologists. Although our study has several limits, it also shows strengths, as well. Data were collected prospectively, although retrospectively evaluated. Surgeons were skilled for each specific approach. Senior residents who were dedicated to urological procedures computed ASA scores, which were however supervised by dedicated consultant. Furthermore, complications were recorded respecting Martin’s criteria according to guidelines [1, 17].

## Conclusions

In PCa patients undergoing RARP surgery, the risk of major postoperative Clavien-Dindo complications increased as the physical status system deteriorated independently by performing or not a PLND and/or large amounts of intraoperative blood lost. The ASA score system was an effective predictor of major Clavien-Dindo complications, which delayed LOHS in RARP surgery. Confirmatory studies are required.

## Supplementary Information

Below is the link to the electronic supplementary material.

Supplementary file1 (DOCX 14.8 KB)
