# Supplementary material for: American Society of Anesthesiologists’ (ASA) Physical Status System and Risk of Major Clavien-Dindo Complications After Robot-Assisted Radical Prostatectomy at Hospital Discharge: Analysis of 1143 Consecutive Prostate Cancer Patients
Source: Indian J Surg Oncol. 2022 Jul 13;13(4):848–57. doi: 10.1007/s13193-022-01577-9 (PMC9845474; doi:10.1007/s13193-022-01577-9)
Supplement: Supplementary file 1 — Supplementary file1 (DOCX 14.8 KB) [file 13193_2022_1577_MOESM1_ESM.docx]

| **Table S1. American Society of Anesthesiologists' (ASA) - Physical Status Classification System** | | | |  |  |  |
| --- | --- | --- | --- | --- | --- | --- |
|  |  |  |  |  |  |  |
|  |  |  |  |  |  |  |
| ***ASA class*** |  | ***Definition*** |  |  |  |  |
|  |  |  |  |  |  |  |
|  |  |  |  |  |  |  |
| **I** |  | A normal healthy patient |  |  |  |  |
|  |  |  |  |  |  |  |
| **II** |  | A patient with mild systemic disease |  |  |  |  |
|  |  |  |  |  |  |  |
| **III** |  | A patient with severe systemic disease |  |  |  |  |
|  |  |  |  |  |  |  |
| **IV** |  | A patient with severe systemic disease that is a constant threat to life |  |  |  |  |
|  |  |  |  |  |  |  |
| **V** |  | A moribund patient who is not expected to survive without the operation |  |  |  |  |
|  |  |  |  |  |  |  |
| **VI** |  | A declared brain-dead patient whose organs are being removed for donor purposes | | |  |  |
|  |  |  |  |  |  |  |
|  |  |  |  |  |  |  |
